# Real-world CAN bus dataset for agricultural tractor block load cycle characterization

**DOI:** 10.1016/j.dib.2026.113244

**Published:** 2026-09-05

**Authors:** Aykut Dana, Orkun Özener, Ali Can Tellioğlu

**Affiliations:** a Test and Validation Department, Tümosan Teknoloji Mühendislik Sanayi Ticaret Anonim Şirketi, Istanbul, Türkiye; b Department of Mechanical Engineering, Yildiz Technical University, Istanbul, Türkiye

**Keywords:** Telemetry, J1939, Ploughing, Mission profile, Engine torque, Duty cycle

## Abstract

Agricultural tractors operate under highly variable load conditions that depend on the field operation, the attached implement, soil characteristics, and operator behavior. Because no standardized, publicly available load cycles exist that reflect this variability, the thermal and durability validation of tractors is commonly performed with boundary-condition scenarios (e.g., continuous maximum torque) that do not represent real field usage. This article presents a dataset of raw, high-resolution Controller Area Network (CAN) bus recordings acquired from an agricultural tractor (Tümosan 81.110 Powershuttle) during real-world operation on fields in the Konya Plain, Türkiye. The tractor was instrumented with a custom-developed CAN-based telemetry system with integrated Global Navigation Satellite System (GNSS) positioning, which continuously streamed the data in real time over a GSM/WebSocket link to a remote server, where the raw frames were decoded into physical values using J1939 signal databases and stored in a time-series database. Approximately one year of operation was recorded at a nominal sampling rate of 10 Hz, covering all activities of the tractor (field work, road transport and idling). From this campaign, the authors filtered the recordings down to the in-field soil tillage segments, which impose the highest engine torque demand, and only these high-load segments are published. The resulting dataset comprises 10 session files, one per worked field, totaling approximately 24.7 h (889,255 samples), so that researchers can focus directly on the most demanding operating condition of the tractor. Each tab-separated value file contains the decoded raw CAN signals. The cyclic alternation between soil-engagement passes under sustained high torque and headland turning events is directly observable in the signals, so that the average duration of working passes under load and of headland turns can be determined. The data can be reused for block load cycle construction, thermal validation on PTO dynamometers, tractor powertrain electrification studies, fuel consumption and productivity analyses and the development of machine state detection and data mining methods for agricultural machinery.

Specifications Table

**Table:** 

Subject	Engineering & Materials science
Specific subject area	Raw CAN bus recordings of an agricultural tractor during soil tillage for load cycle development and thermal validation
Type of data	Table (tab-separated values) Raw (decoded CAN bus signals) Filtered (limited to in-field soil tillage segments)
Data collection	Data were collected with a custom-developed CAN-based telemetry unit (in-house designed PCB with a CAN-compatible GNSS module) installed on a Tümosan 81.110 Powershuttle agricultural tractor (105 hp, maximum engine torque 450 Nm). The unit read the SAE J1939 engine bus and OEM sensor channels at 10 Hz and streamed the raw data in real time over GSM using WebSocket to a remote server, where the frames were decoded into physical values with .dbc/.xml signal databases (PHP back end, InfluxDB storage, Grafana visualization). Approximately one year of operation covering all tractor activities was recorded and then filtered down to the in-field soil tillage segments using rear hitch position, GNSS position, vehicle speed, and engine torque; only these high-load segments are published.
Data source location	Institution: Tümosan Teknoloji Mühendislik A.Ş., Istanbul, Türkiye / Yildiz Technical University, Istanbul, Türkiye Collection sites: agricultural fields in the Konya Plain, Konya, Türkiye
Data accessibility	Repository name: Mendeley Data Data identification number: 10.17632/bbjtjrsxvx.3 Direct URL to data: https://dx.doi.org/10.17632/bbjtjrsxvx.3 Instructions for accessing these data: The repository contains ten tab-separated value (.tab) files, one per recorded field session, together with a README file describing the signal definitions and usage notes. The data are available under a CC BY 4.0 license.
Related research article	*none*

## 1 Value of the Data

• No standardized, publicly available load cycles exist for agricultural tractors, and the few open datasets reflect Central European farm operations. This dataset provides high-resolution (10 Hz), time-synchronized engine, hitch, and aftertreatment signals recorded on fields in the Konya Plain, Türkiye, giving researchers access to real-world tractor load data without the time- and cost-intensive instrumentation and data collection effort.
• The published sessions contain exclusively the in-field soil tillage segments extracted from the complete recording campaign. Because soil tillage is the most demanding operating condition of a tractor, researchers can focus directly on the high-torque operating region without first having to separate field work from transport and idle phases.
• The recordings were obtained from genuine real-user operation rather than controlled experiments. The instrumented tractor was provided to actual farmers, who were informed only that it was a test tractor and used it for their own field work under their own management. Capturing data under these authentic working conditions is considered to yield more representative load and usage information than scripted field trials, in which operator behavior, implement choice, and field selection are constrained by the experiment.
• The cyclic alternation between soil-engagement passes and headland turns is directly observable in the signals, so the data provide the average duration of working passes under sustained high torque and the duration of headland turning events. These quantities are direct inputs for the construction of representative block load cycles, which can be reproduced on a PTO dynamometer for the thermal validation of cooling and aftertreatment systems.
• The combination of engine load parameters (engine speed, actual percent torque, fuel rate) with implement-related signals (rear three-point hitch position, left/right draft sensors, PTO status) and thermal signals (coolant, oil, and aftertreatment system temperatures) allows operating phases to be identified and linked to the thermal response of the engine and exhaust aftertreatment system. Researchers working on tractor electrification can use the torque–speed–time histories to size electric drives, batteries, and cooling systems against realistic mission profiles.
• The combination of wheel-based vehicle speed, actual engine torque, and rear hitch position, used as a proxy for relative implement engagement depth, recorded synchronously in each session enables researchers to characterize the influence of travel speed and implement depth on engine torque demand during soil tillage.
• The provided data can be combined through multi-sensor fusion to classify operational phases (e.g., working phase / turning phase); after filtering out short-duration signal fluctuations, phase-level features (duration, load, and speed averages) can be extracted for each task and normalized; a distance/similarity-based analysis can then be used to identify the most representative high-load task; and by averaging the phase statistics of the selected task, a repeated block load cycle can be defined.
• The per-field session files are suitable for developing and testing automated data mining methods for agricultural machinery, including machine state detection, operation classification, fuel consumption and productivity analysis, and anomaly detection on CAN bus data.

## 2 Background

Thermal performance validation of agricultural tractors is conventionally based on boundary-condition scenarios such as continuous maximum torque, maximum power, regeneration, and hot shutdown. While suitable for probing extreme limits, these scenarios do not represent real field operation, in which the load alternates cyclically between sustained high-torque working passes and low-load maneuvering phases. Publicly available real-world tractor datasets and reference working cycles [[1], [2], [3]] reflect Central European farm operations and specific tractor fleets, standardized cycles such as OECD Code 2 focus on engine emissions rather than whole-vehicle usage, and commercial test procedures such as the DLG PowerMix [4] are not accessible for research purposes [2].

Open, high resolution CAN bus datasets for tractor load characterization remain scarce. Götz et al. [1] provide 862 h of telemetry, including 354 h of fieldwork across 143 fields, from five Fendt tractors ranging from 77 to 240 kW, sampled at 10 Hz and segmented by tractor and implement. This breadth is distributed across five platforms, so the volume of data available per individual tractor is comparatively limited. The Fendt 211, the platform closest in rated power to the present tractor, is represented by only four published field sessions. The present dataset instead concentrates ten field sessions on a single tractor and implement pairing, a Tumosan 81.110 Powershuttle performing soil tillage, providing substantially more per tractor session data for duty cycle characterization. Table 1 compares per field torque statistics computed identically for both datasets. Overall average torque across the ten present sessions ranges from 34.46% to 72.11%, while across the four Fendt 211 sessions it ranges from 20.02% to 37.97%. Averaged across all sessions, the present dataset exhibits a substantially higher torque density than the Fendt 211 subset, with a mean overall torque of 55.95% against 28.70%. This gap reflects the heavier draft demand of soil tillage relative to power harrowing and precision seeding, together with the selection of work sequences with relatively high average torque for release in the present dataset.

**Table 1:** Comparison of per-field engine torque statistics between the present dataset (Tumosan 81. 110 Powershuttle) and the Fendt 211 subset of Götz et al. [1]. Duration is derived from sample count × 100 ms per row for both datasets; in-work average torque uses HtcPositionSensPerc ≤ 60% (implement lowered) for the present dataset and the Status = "working" label provided by Götz et al. [1] for the Fendt 211 subset.

Tractor	Field	Total Duration	Duration (h)	In-Work Avg. Torque (%)	Overall Avg. Torque (%)
Tümosan 81.110	FIELD_1	1 hr 20 min	1.34	44.60%	34.46%
Tümosan 81.110	FIELD_2	3 hr 55 min	3.92	72.71%	67.48%
Tümosan 81.110	FIELD_3	1 hr 31 min	1.51	64.20%	59.48%
Tümosan 81.110	FIELD_4	1 hr 58 min	1.96	62.11%	54.59%
Tümosan 81.110	FIELD_5	3 hr 40 min	3.67	62.44%	55.45%
Tümosan 81.110	FIELD_6	1 hr 34 min	1.56	70.87%	60.34%
Tümosan 81.110	FIELD_7	1 hr 47 min	1.79	54.91%	50.96%
Tümosan 81.110	FIELD_8	5 hr 2 min	5.04	71.42%	62.20%
Tümosan 81.110	FIELD_9	1 hr 26 min	1.44	49.46%	42.39%
Tümosan 81.110	FIELD_10	2 hr 29 min	2.48	81.56%	72.11%
Fendt 211 [1]	Power harrowing, Field 1	1 hr 23 min	1.38	40.31%	37.97%
Fendt 211 [1]	Power harrowing, Field 2	0 hr 43 min	0.71	43.55%	35.74%
Fendt 211 [1]	Precision air seeding, Field 1	0 hr 24 min	0.40	17.58%	20.02%
Fendt 211 [1]	Precision air seeding, Field 2	0 hr 16 min	0.26	22.56%	21.07%

The lack of representative load cycles has also been identified as a major obstacle for tractor electrification studies [5,6], although real-world load measurements are increasingly used to dimension electric and hybrid tractor powertrains [[7], [8], [9]], and hybrid solutions for agricultural vehicles are being assessed from the users’ standpoint over their life cycle [10].

This dataset was compiled to characterize the loads acting on the tractor during soil tillage, the most demanding operating condition of agricultural tractors. The recordings provide a real-world basis for the construction of representative block load cycles—for example, by selecting representative operating segments using normalization and distance metrics—which can be reproduced on a PTO dynamometer for the thermal performance evaluation of cooling and exhaust aftertreatment systems. The published files allow such derivations to be reproduced, extended, or applied to other use cases.

## 3 Data Description

The repository contains ten recorded soil tillage sessions as tab-separated value files (.tab) together with a README file documenting the signal definitions. Each file holds the decoded raw CAN bus signals of one continuous soil tillage session on a single worked field, filtered out of an approximately one-year recording campaign as described in the section Experimental design, materials and methods. The files follow the naming convention FIELD_{id}_TUMOSAN_81110P_TILLAGE_OP_RAW.tab, where the file name encodes the field identifier, the tractor model (Tümosan 81.110 Powershuttle), the operation (soil tillage), and the raw nature of the data. All ten sessions were recorded on different fields in the Konya Plain, Türkiye, and together comprise approximately 24.7 h (889,255 samples) of in-field soil tillage operation.

Each file contains a header row followed by the time-series data on a uniform 10 Hz grid. The first column, time_ms, is a relative timestamp in milliseconds that starts at zero in every file and increases in steps of 100 ms; the absolute recording date and time are not disclosed, in order to anonymize the dataset. The remaining columns contain the decoded raw CAN signals listed in Table 2. The engine signals, DCU (Dosing Control Unit) and Tractor ECU signals follow the SAE J1939 standard and are identified by their Suspect Parameter Numbers (SPN) and Parameter Group Number (PGN). The Electronic Lift Controllerand keypad signals are read from OEM-specific sensor channels. Engine torque is provided as a percentage of the engine reference torque (SPN 513); the absolute torque in Nm is obtained by multiplying this percentage by the maximum engine torque of 450 Nm. Datasets and graphs for the torque and power curves showing the tractor engine's performance characteristics are available for open access in the study's data repository. Despite the limited precision of the custom-built telemetric data acquisition system's GNSS module, the data provides a reliable baseline for tracking general tractor movements. For privacy considerations, GNSS data was included in the dataset after being normalized to a fixed (0,0) latitude/longitude reference. The signal values are provided as decoded raw values without smoothing or interpolation; depending on the operating state, individual signals may temporarily hold default or initialization values (e.g., zero) during signal dropouts and engine start, and such samples are retained so that the user can filter them according to the intended analysis.

**Table 2:** Description of the signals contained in the FIELD_{id}_TUMOSAN_81110P_TILLAGE_OP_RAW.tab files. The source address designations used in this table follow the SAE J1939 standard: source address 0 (Engine #1) corresponds to the engine ECU, source address 240 to the Tractor ECU, source address 35 (Hitch Control) to the electronic lift control panel, source address 61 (Exhaust Emission Controller) to the DCU (Dosing Control Unit) and source address 144 (Keypad Buton Device) to the keypad.

Signal	Source Address	Unit	PGN/SPN	Description
time_ms	"-/-"	ms	–	Relative timestamp in milliseconds; starts at zero in every file and increases in uniform steps of 100 ms (10 Hz). The absolute recording date and time are not disclosed for anonymization.
ActualEngine_PercTorque	0 - Engine #1	%	61444/513	Actual engine torque, reported as a percentage of the engine reference (maximum) torque. The absolute torque in Nm is obtained by multiplying this percentage by the maximum engine torque of 450 Nm (e.g., 80% corresponds to 360 Nm).
EngineSpeed	0 - Engine #1	rpm	61444/190	Actual engine rotational speed.
EngOilTemp1	0 - Engine #1	°C	65262/175	Temperature of the engine lubrication oil.
EngOilPress	0 - Engine #1	kPa	65263/100	Gauge pressure of the oil in the engine lubrication system.
EngCoolantTemp	0 - Engine #1	°C	65262/110	Temperature of the engine coolant.
EngFuelRate	0 - Engine #1	L/h	65266/183	Amount of fuel consumed by the engine per unit time.
WheelBasedVehicleSpeed	240 - Tractor ECU	km/h	65265/84	Vehicle speed calculated from the measured wheel speed.
HtcDraftSens1Perc	35 - Hitch Control	%	OEM	Draft force at the left lower link of the rear three-point hitch, as a percentage of the sensor measuring range. Rare out-of-range readings near the channel limits may occur and should be treated as invalid samples.
HtcDraftSens2Perc	35 - Hitch Control	%	OEM	Draft force at the right lower link of the rear three-point hitch, as a percentage of the sensor measuring range. Rare out-of-range readings near the channel limits may occur and should be treated as invalid samples.
HtcPositionSensPerc	35 - Hitch Control	%	OEM	Measured position of the rear three-point hitch (low values: implement lowered/in work; high values: implement raised/out of work). Used to separate working passes from headland turns. Values slightly outside the 0–100% range may occur due to sensor calibration tolerance.
TransferCaseStatus	240 - Tractor ECU	bit	64899/3645	Engagement status of the four-wheel-drive transfer case (0: disengaged, 1: engaged).
PTO_Status	240 - Tractor ECU	bit	65091/2408	Engagement status of the rear power take-off (PTO) shaft (0: disengaged, 1: engaged). PTO is disengaged for ploughing (draft-only) and engaged for rotary tilling (PTO-driven), so this signal distinguishes the two operations.
ATSTemp	61 - Exhaust Emission Controller	°C	64830/4360	Exhaust gas temperature measured at the aftertreatment system (ATS).
KeypadButton	144 - Keypad Buton Device	–	OEM	Operator-declared implement selection entered via the in-cab keypad (1: Unallar reversible plough, 2: Hisarlar Alabora 300 rotary tiller). Constant within each session file.
FuelLevel1	240 - Tractor ECU	%	65276/96	Indicates the instantaneous fuel amount in the fuel tank as a percentage (%).
Longitude	"-/-"	-	-	Normalized longitude positioning data, offset relative to a fixed (0,0) reference coordinate to ensure spatial data confidentiality while retaining relative tractor kinematics.
Latitude	"-/-"	-	-	Normalized latitude positioning data, offset relative to a fixed (0,0) reference coordinate to ensure spatial data confidentiality while retaining relative tractor kinematics.

**Fig. 1:**
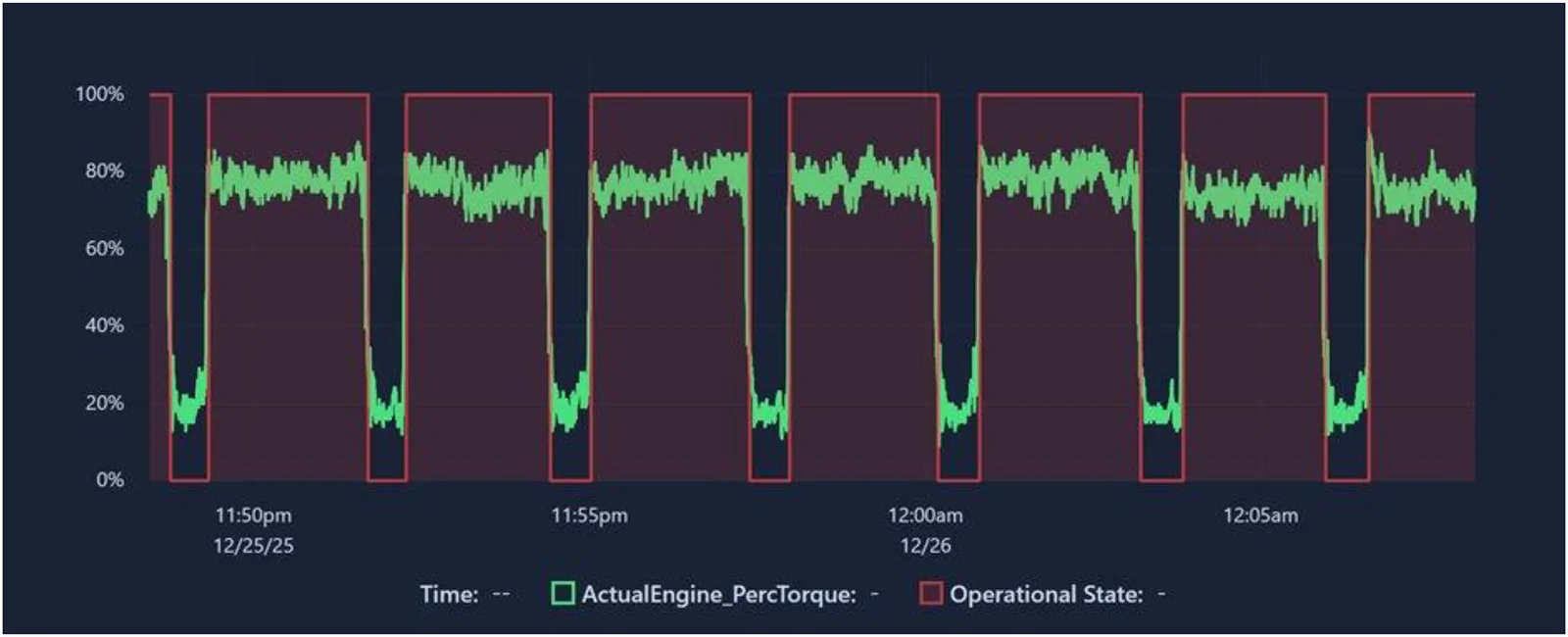
Example of real-time engine torque percentage (%) and operational states (100% means ploughing and 0% means headland turning) during field operations.

The published sessions contain in-field soil tillage operation; road transport, idling, and other activities recorded during the campaign were removed during curation, while short in-field repositioning maneuvers between working passes are retained. This allows researchers to focus directly on the most demanding operating condition of the tractor. Within each session, the cyclic alternation between soil-engagement passes and headland turning events is directly observable from the rear three-point hitch position signal in combination with engine torque and vehicle speed: the working passes take place under sustained high torque and are separated by short headland turns, during which the implement is lifted, the torque demand drops, and the engine speed rises. From these signals, the average duration of working passes under load and the duration of headland turning events can be determined per field. The PTO and transfer case status signals indicate the powertrain configuration during the operation. The torque required for soil tillage is governed primarily by soil texture; the test fields are located in the Konya Plain, whose agricultural soils are predominantly semi-arid, calcareous, alluvial clay-loam to lacustrine clay soils [11], which is useful context when interpreting the recorded load levels. Fig. 1 illustrates the load behavior for a representative portion of a tillage session, showing the measured engine percent torque together with the derived operational state, defined here as in-work (100%) when the rear hitch is in the lowered/working position and out-of-work (0%, headland turn) when it is raised.

Two soil tillage implements were used during the recordings: a mounted reversible plough and a rotary tiller. The plough is a draft-type implement pulled through the soil without power take-off (PTO) drive, so the PTO is disengaged (PTO_Status = 0) throughout the ploughing sessions; the rotary tiller is PTO-driven, so the PTO is engaged (PTO_Status = 1) during the rotary tilling sessions. In addition, the operator-declared implement selection is included in every file as the KeypadButton channel (1: plough, 2: rotary tiller), so both the KeypadButton and the PTO_Status signals directly distinguish the two operations. Eight of the ten sessions are ploughing and two (FIELD_7 and FIELD_10) are rotary tilling. Table 3 lists the implement assigned to each worked field together with the corresponding number of samples and recorded duration. The four-wheel-drive transfer case is engaged (TransferCaseStatus = 1) during essentially the entire dataset, as expected for heavy tillage work.

**Table 3:** Implement, operation, and recorded amount of data for each worked field*.*

Field	Operation	Implement (make / model)	Samples	Duration [h]
FIELD_1	Ploughing	Unallar reversible plough (5-furrow, 12″)	48,247	1.34
FIELD_2	Ploughing	Unallar reversible plough (5-furrow, 12″)	140,948	3.92
FIELD_3	Ploughing	Unallar reversible plough (5-furrow, 12″)	54,429	1.51
FIELD_4	Ploughing	Unallar reversible plough (5-furrow, 12″)	70,588	1.96
FIELD_5	Ploughing	Unallar reversible plough (5-furrow, 12″)	132,103	3.67
FIELD_6	Ploughing	Unallar reversible plough (5-furrow, 12″)	56,056	1.56
FIELD_7	Rotary tilling	Hisarlar Alabora 300 rotary tiller (3 m, 60 blades)	64,448	1.79
FIELD_8	Ploughing	Unallar reversible plough (5-furrow, 12″)	181,488	5.04
FIELD_9	Ploughing	Unallar reversible plough (5-furrow, 12″)	51,754	1.44
FIELD_10	Rotary tilling	Hisarlar Alabora 300 rotary tiller (3 m, 60 blades)	89,194	2.48

**Fig. 2:**
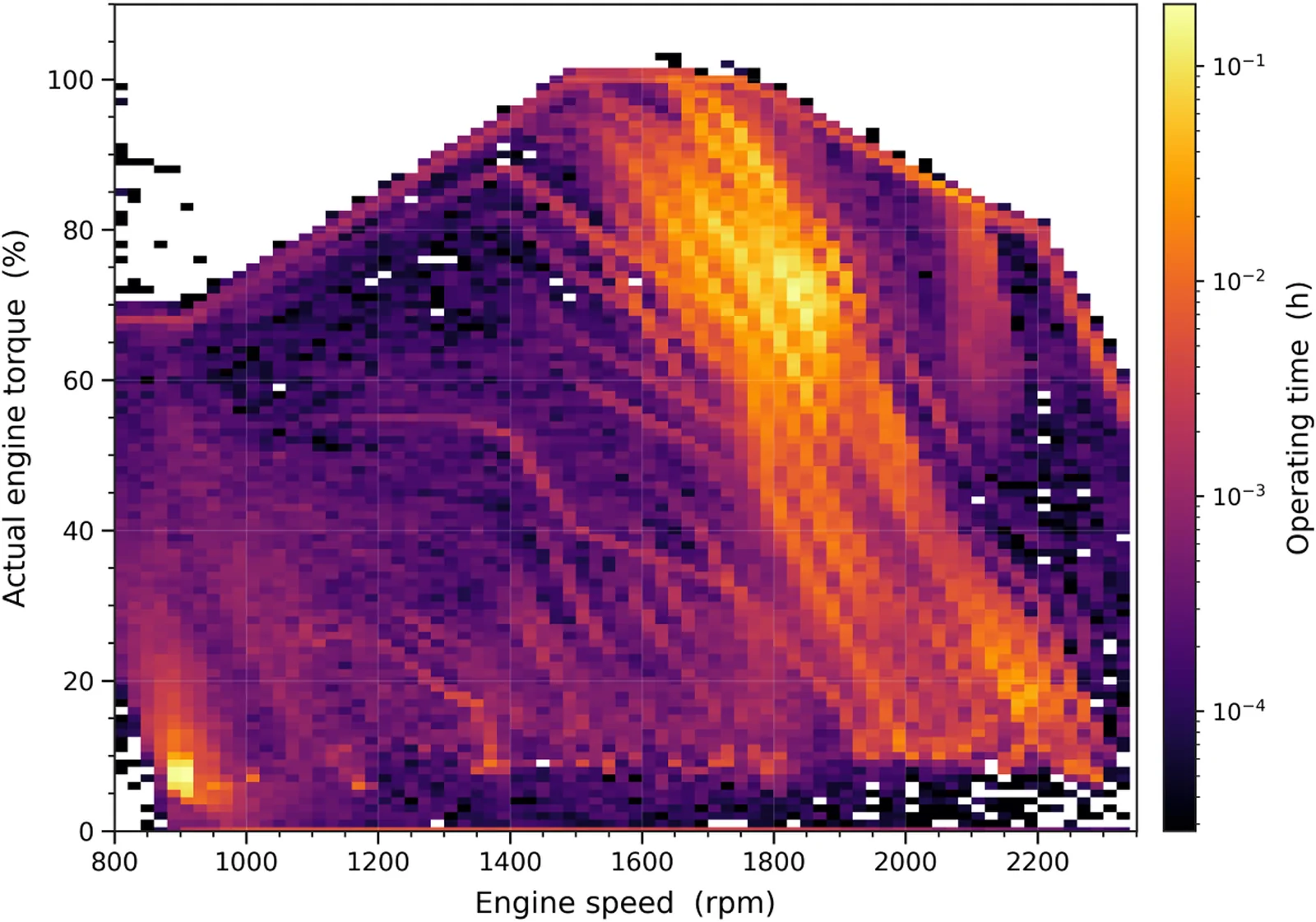
Operating-point density of the complete dataset in the engine speed–torque plane. The two-dimensional histogram aggregates all ten soil tillage sessions; the color encodes the cumulative operating (residence) time in each 20 rpm × 1% bin on a logarithmic scale. Actual engine torque is reported as a percentage of the 450 Nm reference torque (SPN 513). The operating points concentrate in the high-load tillage region around 1600–1900 rpm at 70–85% torque (≈ 315–380 Nm)*.*

Considered across the complete dataset, the engine operating points are strongly concentrated in the high-load region characteristic of soil tillage. Fig. 2 shows the two-dimensional residence-time density of all ten sessions in the engine speed–actual torque plane.The distribution exhibits a dense load band that rises from the idle region near 900 rpm to a pronounced maximum around 1600–1900 rpm at 70–85% of the 450 Nm reference torque (approximately 315–380 Nm), consistent with the sustained high-torque demand imposed by ploughing and rotary tilling. This aggregate view confirms that the published segments capture the most demanding operating condition of the tractor and provides the load distribution required for the construction of representative block load cycles.

**Fig. 3:**
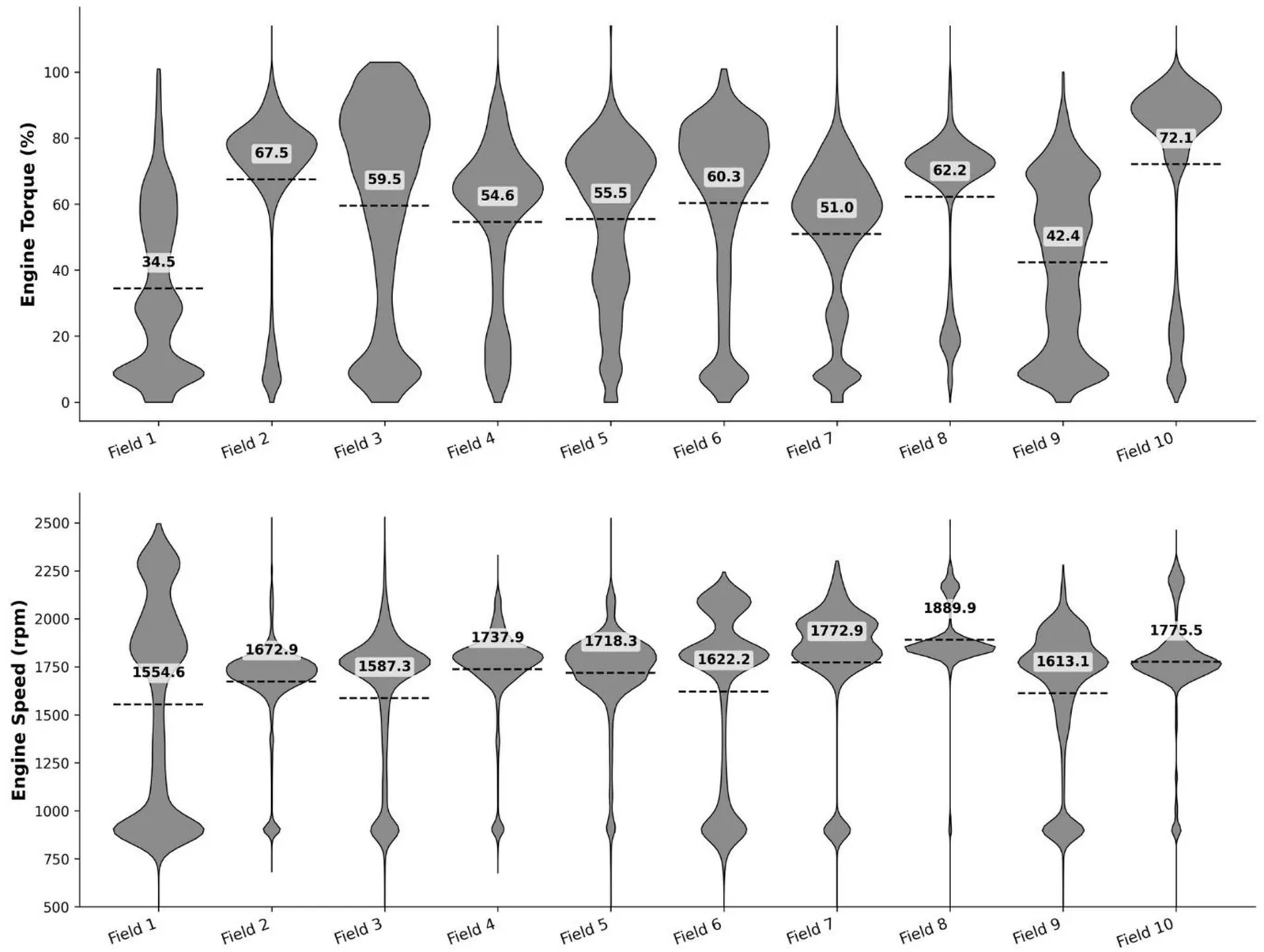
Engine torque (%) and engine speed (rpm) distributions for each of the ten soil tillage sessions. Violin width represents sample density; dashed lines and boxed values mark the per-field mean.

Fig. 3 disaggregates this distribution by field, showing the engine torque and engine speed spread recorded in each session individually. Field-level mean torque ranges from 34.5% to 72.1% of the 450 Nm reference torque (155–324 Nm), reflecting differences in soil resistance, implement, and operator behavior across fields. This session-level variability provides the basis for selecting representative segments for block load cycle construction.

## 4 Experimental Design, Materials and Methods

### 4.1 *Telemetry and data acquisition system*

Field data were collected using a custom-developed Controller Area Network (CAN)-based telemetry system with integrated Global Navigation Satellite System (GNSS) positioning. An in-house designed electronic control unit and a GNSS module with CAN data output were installed on a Tümosan 81.110 Powershuttle agricultural tractor with a rated engine power of 105 hp, enabling simultaneous acquisition of engine sensor data and positional information. The system is compatible with any vehicle equipped with a CAN bus communication interface. The acquired channels comprise the SAE J1939 engine signals and OEM sensor channels listed in Table 2, including engine speed, actual percent torque, fuel rate, temperatures and pressures, vehicle speed, rear three-point hitch position and draft forces, PTO and transfer case status, and aftertreatment system temperature. The electronic lift control unit (source address 35) is a Mita M-75, and the in-cab keypad (source address 144) used for operator-declared implement selection is a Makersan MO 405 H3X.

**Fig. 4:**
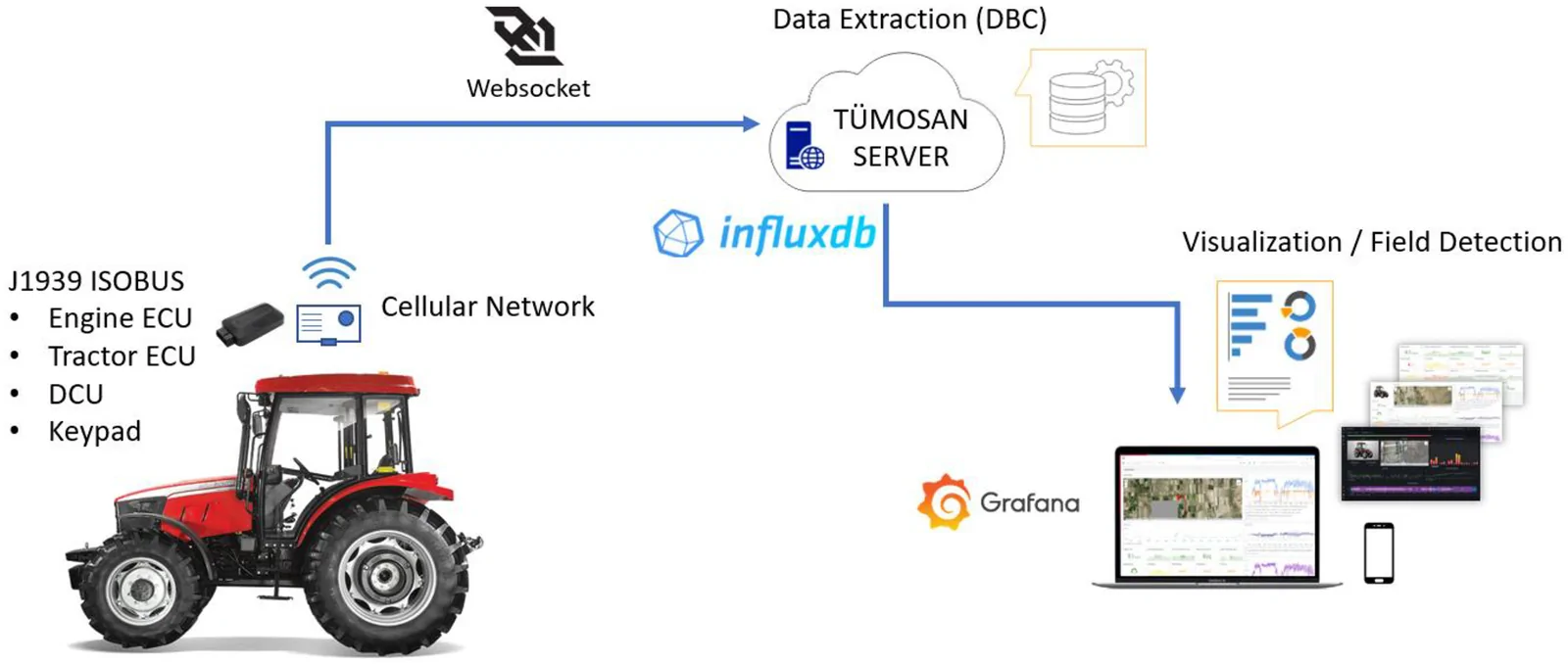
Schematic representation of the telemetric data acquisition system*.*

The data were transmitted in real time to a remote server over the GSM mobile network. Transmission was implemented with WebSocket technology, which provides bidirectional (full-duplex) communication between the on-board client and the server and thus enables low-latency, uninterrupted data streaming without repeated request–response cycles. On the server side, a PHP-based application received and processed the incoming data stream. The raw CAN frames were decoded into physical engineering values in real time using .dbc and .xml signal database files, which define the scaling factors, offsets, value ranges, and physical units of each signal. The decoded time series were stored in the open-source time-series database InfluxDB, and Grafana was integrated for real-time monitoring, historical analysis, and visualization. All software components were implemented with open-source solutions; the embedded data acquisition software was developed in C++ and the server-side processing layer in PHP. The overall architecture of the telemetric data acquisition system is shown in Fig. 4.

### 4.2 CAN frame decoding procedure

The server-side decoding of the incoming CAN stream comprises three functional layers, from raw frame capture to storage of the decoded physical quantities. In the first layer, the on-board telemetry unit monitors, over a single physical CAN bus, both SAE J1939-compliant Engine ECU, Tractor ECU, DCU signals and manufacturer-specific (OEM) messages from the Electronic Lift Control and Keypad, and logs each frame together with its CAN identifier (CAN ID), raw data bytes, and a single timestamp assigned at the time of capture. In the second layer, these raw frames are structured in JSON format, without any physical conversion, and transmitted to the server over the WebSocket link described above. In the third layer, the server-side decoding engine loads the DBC (CAN database) file corresponding to the device configuration and maps each frame to its message definition through exact CAN ID or J1939 Parameter Group Number (PGN) matching.

During physical conversion, the relevant bits are extracted from the raw data bytes by bit shifting and bit masking, using the start bit and bit length parameters defined for each signal in the DBC file; for multi-byte signals, the constituent bytes are combined into a raw integer value according to the specified byte order (little-endian/big-endian). This raw value is then converted to the corresponding physical quantity using the linear relationship given in Eq. (1):

(1) PhysicalValue=(RawValue×ScaleFactor)+Offset

For categorical signals conveying status or mode information, a lookup table is additionally applied after this linear conversion to map the raw codes to their corresponding categorical values. Frames for which no matching CAN ID/PGN is found, or whose byte length does not match the expected value, are treated as invalid during decoding and discarded, thereby preventing corrupted or incomplete data packets from being included in the dataset.

Temporal consistency of the system is ensured by preserving, unaltered from acquisition to storage, the single timestamp generated for each frame at the moment of capture, and by resampling all J1939 and OEM signals — despite their differing native broadcast periods — to a fixed 10 Hz sampling rate. This approach aligns signals originating from different subsystems (Engine ECU, Tractor ECU, DCU, Electronic Lift Control and Keypad) on a common time axis, yielding a temporally consistent dataset without the need for any additional resampling or interpolation step.

### 4.3 Field data collection

The instrumented tractor was assigned to operators and used in regular agricultural work under real operating conditions on farms in the Konya Plain over approximately one year, spanning a full agricultural cycle. The data were recorded at a nominal sampling rate of 10 Hz and cover all activities of the tractor, including field operations under different load levels, road transport between fields, and idle phases. In addition to the automatically acquired signals, a user interface allowed the operator to declare the implement attached during the operation; this operator-declared implement information was logged together with the telemetry stream and used during data curation to identify the performed operation and is included in the published files as the KeypadButton channel. Two soil tillage implements were used: a mounted reversible plough (Unallar, five furrows, 12-inch furrow width, suited to 90–110 hp tractors) for the ploughing sessions and a PTO-driven rotary tiller (Hisarlar Alabora 300, 3 m working width, 60 blades) for the rotary tilling sessions.

**Fig. 5:**
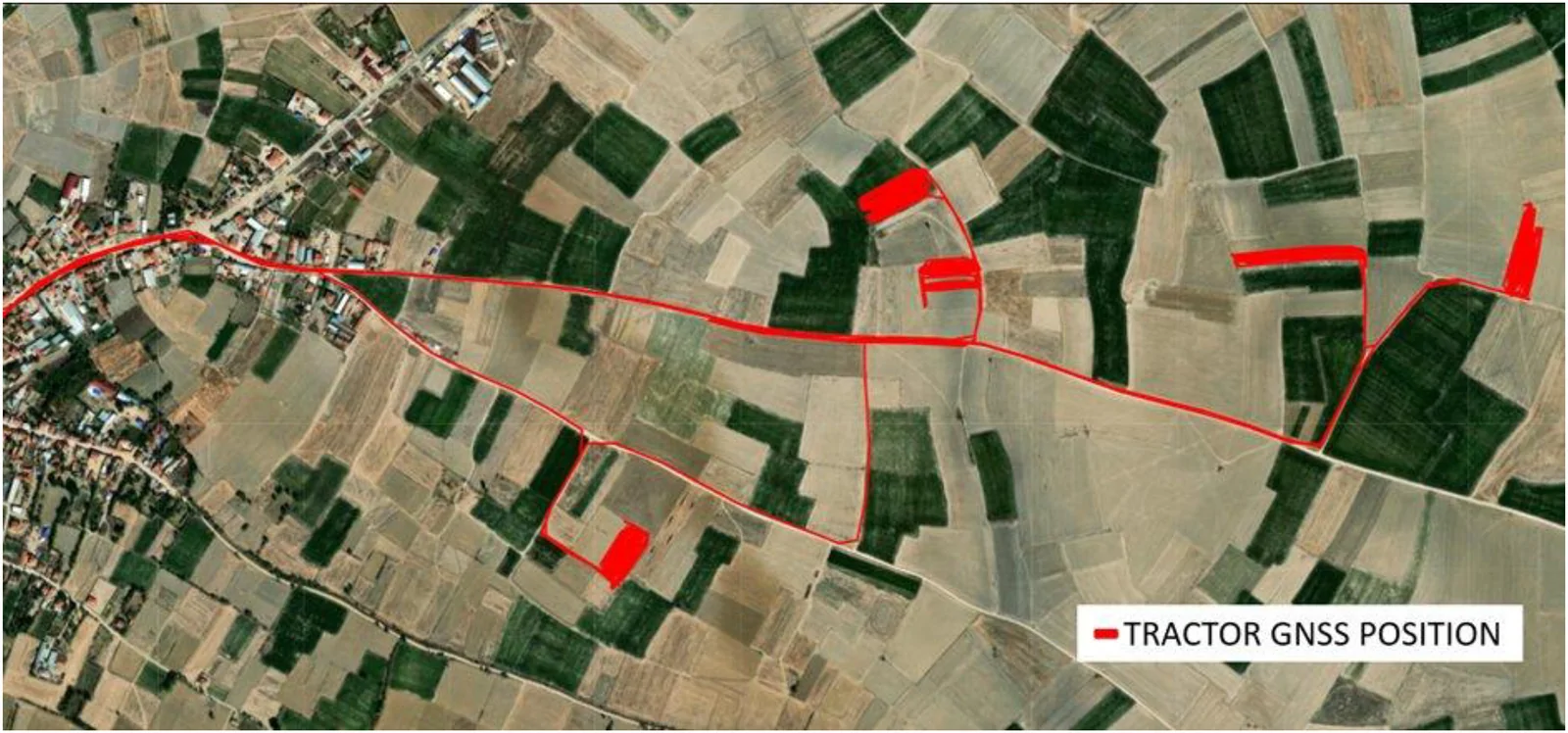
Tractor route based on telemetric positioning data, showing movements between different fields and return paths*.*

The tests were carried out with genuine farmers under real working conditions. The instrumented tractor was made available to the farmers, who operated it for their own field work according to their own practice; they were aware that it was a test tractor equipped for data collection, and only machine operating data were recorded, with no personal data and no disclosure of the absolute recording dates. Collecting data in this way, rather than through controlled or scripted farming trials, was intended to capture more realistic real-user operating conditions, in which the operator behavior, implement choice, and field selection are not constrained by the experiment. Fig. 5 shows the resulting tractor route reconstructed from the telemetric positioning data, including the movements between the different worked fields and the return paths.

### 4.4 Extraction of the soil tillage segments

The published dataset is a curated subset of the approximately one-year recording campaign, reduced to approximately 24.7 h (889,255 samples) through several successive stages of selection rather than a single filtering step. These stages reflect, in turn, the restriction to soil tillage operations among the many implements used during the campaign, the exclusion of segments affected by unstable mobile network transmission, a preference for temporally continuous operations, and the exclusion of transport driving. Each of these considerations is described below, followed by the quantitative criteria used to delineate the boundaries of each retained session.

Although the acquisition campaign spanned approximately one year, the total engine operating time within this period was approximately 358 h.

Among the many different implements used during these 358 h of operation, such as seed drills, chisel ploughs, cultivators, rotovators, spraying and fertilizing equipment, harvesting machinery, balers, and trailers, only the segments corresponding to soil tillage operations were retained. These segments were identified with the help of a CAN-output keypad placed inside the cabin, on which the operator selected the key corresponding to the implement in use; the resulting implement information was logged together with the telemetry stream.

Difficulties in GSM mobile network access also constrained which segments could be included. In field areas far from residential zones and base stations, the remote data acquisition system could not operate stably, and instantaneous interruptions in the data stream occurred frequently. Operating data in which data integrity was ensured were identified through subjective evaluation and included in the study.

For the sake of operational continuity, soil tillage operations exhibiting as much continuity as possible were included through subjective evaluation, while segments containing long-duration stops due to implement hitching, unhitching, or adjustment were excluded.

Even when a soil tillage implement remained attached to the tractor — that is, even when a soil tillage implement was selected on the keypad — the segments in which the tractor was in transport between the farm and the field were excluded, since the main objective of this study is to present data from soil tillage operations, in which the tractor operates under low speed and high torque.

Guided by these considerations, the authors extracted the segments in which the tractor was operating inside a field and performing soil tillage (ploughing or rotary tilling) by jointly considering the rear three-point hitch position (implement in the lowered/working position, indicating engagement with the soil), the GNSS position (tractor located within a field rather than on the road network), the vehicle speed (within the typical tillage working-speed range of roughly 4–12 km/h), and the engine torque (sustained high load), an approach comparable to field–road trajectory segmentation methods [12] and CAN bus based mission-profile analyses [13]. Headland turning events within a field, identified by abrupt increases in the hitch position together with a drop in torque and a rise in engine speed, were retained as part of the session, whereas idling and other non-tillage activities were removed. The data of each identified soil tillage session were exported into a separate tab-separated value file named with the field identifier, the tractor model, the operation, and the raw nature of the data (FIELD_{id}_TUMOSAN_81110P_TILLAGE_OP_RAW.tab), resulting in ten session files comprising approximately 24.7 h (889,255 samples) in total. The absolute GNSS latitude/longitude coordinates were removed from the published files for privacy reasons; instead, each session includes GNSS coordinates normalized relative to a fixed (0,0) latitude/longitude reference point corresponding to the start of that session, allowing the tractor's relative in-field trajectory to be reconstructed without disclosing its absolute geographic location. The signal values are provided as decoded raw CAN values; no resampling beyond the native 10 Hz logging grid, smoothing, interpolation, or removal of default/initialization values was applied, so that users retain full control over subsequent filtering.

Beyond meeting the hitch-position, GNSS, speed, and torque criteria described above, the ten published sessions were further required to exhibit telemetry continuity (excluding intervals affected by GSM signal loss in remote field areas) and operational continuity (excluding intervals with prolonged stoppages for implement attachment, detachment, or adjustment). Consequently, the published subset does not constitute an exhaustive extraction of every qualifying tillage segment recorded during the one-year campaign, but a curated selection satisfying these additional data-quality criteria. The dataset is thus intended to characterize high-load soil tillage operation specifically, and should not be interpreted as representing the tractor's complete real-world duty cycle, which would also encompass transport, idling, and other field operations.

## Limitations

The recordings were obtained from a single tractor (Tümosan 81.110 Powershuttle, 105 hp) operating on fields in the Konya Plain, Türkiye; the load levels and durations therefore reflect the implements, soil conditions, and operator behavior of this specific operation and may differ for other tractor sizes, regions, and farm structures. In particular, the torque demand of soil tillage depends strongly on soil texture and moisture, so the recorded loads should be interpreted in the context of the predominantly calcareous clay-loam to clay soils of the Konya Plain [11]. The published dataset contains only the extracted soil tillage segments; road transport, idling, and other field operations recorded during the campaign are not included, so the share of time between field work and other activities cannot be derived from the published files.

It should be noted that the published dataset does not capture a full-lifecycle mission profile of the tractor. However, this focused coverage was a deliberate methodological choice. Research and development efforts typically prioritize the most critical operating phases, where the vehicle experiences peak structural and thermal stresses. Focusing on high-torque soil tillage provides high-resolution data for these critical stages. This approach aims to streamline development cycles and reduce the need for prolonged, costly field data acquisition. Nevertheless, supplementary datasets covering omitted operational modes, broader power ratings, and quantified soil parameters remain an important focus for future work.

The absolute recording dates are not disclosed for anonymization. Due to the hardware architecture of the custom-built telemetric data acquisition system, the spatial precision of the integrated GNSS module is relatively limited, serving primarily to capture macroscopic vehicle trajectories rather than high-resolution kinematics. Furthermore, to enforce spatial data confidentiality, all geographic coordinates (latitude and longitude) have been offset and normalized relative to a fixed (0,0) reference origin, which intentionally omits absolute global positioning from the dataset while preserving relative displacement patterns.Consequently, the data cannot be combined with external date- or location-dependent sources. Individual signals may temporarily hold default or initialization values (e.g., zero in temperature or pressure channels) during signal dropouts and engine start, and the draft and hitch-position channels occasionally report values outside their nominal ranges; these samples are retained in the raw files and must be filtered by the user.

## CRediT Author Statement

**Aykut Dana:** Conceptualization, Methodology, Software, Investigation, Data curation, Formal analysis, Visualization, Writing – original draft. **Orkun Özener:** Supervision, Methodology, Writing – review & editing. **Ali Can Tellioğlu:** Investigation, Data curation, Validation, Visualization.

## Ethics Statement

The authors have read and followed the ethical requirements for publication in Data in Brief and confirm that the current work does not involve human subjects, animal experiments, or any data collected from social media platforms.
